# Report of Cases in the Medical Clinic

**Published:** 1855-11

**Authors:** J. G. Westmoreland

**Affiliations:** Professor of Materia Medica


ATLANTA

### Medical and Surgical Journal.

VOL. I.] NOVEMBER, 1855. [No. 3.

### ORIGINAL COMMUNICATIONS.

ARTICLE I.—*Report of Cases in the Medical Clinic during the Course of Lectures of the present year in the Atlanta Medical College.* By J. G. WESTMORELAND, M. D., Professor of Materia Medica.

In several successive numbers of the Journal will appear a succinct history of some of the most interesting cases presented to the class during the session. Such only will be noticed as, from their character, make up a variety of the diseases occurring in this city and vicinity.

The importance of clinical instruction is derivable more from the variety of cases presented than from their magnitude; hence, cases so selected as to afford instruction on the greater number of diseases are preferable. Affections, indeed, which rarely if ever prove fatal have to be treated by physicians, and a knowledge of their cause, nature, and treatment, is as indispensable to the success of a young practitioner as that of the more serious affections.

We shall commence with the report of cases which afford means for the study of cutaneous diseases. These, though not generally dangerous, prove more troublesome to the patient, and perplexing to the physician, than many diseases less apt to terminate favorably.

CASE 1.—A child, aged about six months, the subject of vesicular eruption for two months or more, was brought into the College Clinic about the first of June last. The vesicles were more abundant about the head neck and chest. The eruption appeared first in patches of distinct vesicles, which, on maturing, became confluent, and discharged the milk-like contents, making a continuous scab for several inches. An intolerable itching was the most distressing symptom in the case of this little sufferer. So harassing was this sensation that for hours it could be quieted only by rubbing the parts affected. Successive crops or patches of the eruption made their appearance in the vicinity of the original, as desquamation took place, sometimes even in the same tender part that had just cast off scab.

In regard to the true nature of this case various conjectures of Tertiary Syphilis, &c., were entertained, but from all the symptoms and circumstances connected with the case it was evidently Eczema, a cutaneous affection belonging to the class of vesicular eruptions. In this case the symptoms were of an unusually aggravated character; and, as in most of the non-febrile eruptive diseases, this case required ocular investigation to determine its true nature.

The irritation produced by the severity and repeated appearance of the eruption extended finally to the subcutaneous cellular tissue, producing numerous abscesses or furuncles of considerable size. The exhaustion and irritability from these upon a subject enfeebled by the tormenting eruption for three or four months, came well nigh destroying the patient. With supporting treatment, however, and by calming the irritability of the nervous system, the life of the child was saved.

CASE 2.—A boy, aged about 8 years, general health good, was attacked about the first of July with vesicular eruption on the face, particularly on the eyebrow and on the cheek near the eye. At first the appearance was very similar to that produced by sprinkling the surface with boiling water. On the rupture of the vesicles, in several places, the surface for an inch or more in diameter, exhibited a continuous scab of concreted exudation. A fresh crop of vesicles would appear in the immediate neighborhood of these, sometimes before and sometimes after the desquamation of the former patches.

In the treatment of this disease no plan can be given which is applicable to all cases; and, indeed, perhaps a majority of the cases that occur require no treatment at all. It is only necessary in such to know the character and tendency of the affliction, that the anxiety of the patient and friends may be allayed. When treatment is required at all, each particular case affords symptoms by which the local and general applications must be determined. In the cases given above, one required no prescription whatever, while the other demanded constant applications to allay the pruritus and irritability of the parts, and general constitutional remedies in order to support and quiet the system under the heavy draft made upon it by so large an irritable surface. It is not usual for fever to be developed in an ordinary case of eczema ; if, however, from the extent of surface involved in the eruption, or excessive excitement in the part, fever should result, such general treatment as will naturally suggest itself to one acquainted with inflammatory and febrile affections will be required.

It is sometimes very difficult to make a correct diagnosis of diseases, such as the one under consideration. It is, in fact, impossible in some instances, when the disease has progressed for some time before the physician sees it, to determine, from the appearance of the part, whether it originated in a pustular or vesicular eruption. A complete history of the case—as regards time and manner of attack, &c.—is necessary generally in order to determine the nature of the disease.

Though eczema generally has a specific time to run its course, which is only a few weeks, it sometimes, as in case one above, is protracted to as many months. This variation in regard to duration is not pecular to this disease ; for in those affections of a more specific origin and character, whose duration is generally very exactly known, from accidental local inflammation are greatly prolonged.

